# Quantitative analysis of lipids: a higher-throughput LC–MS/MS-based method and its comparison to ELISA

**DOI:** 10.4155/fsoa-2016-0067

**Published:** 2017-01-16

**Authors:** Adarsh S Gandhi, David Budac, Tanzilya Khayrullina, Roland Staal, Gamini Chandrasena

**Affiliations:** 1 Molecular Pharmacology, Bioanalysis & Operations, Lundbeck Research USA, 215 College Road, Paramus, NJ, USA; 2 Neuroinflammation Disease Biology Unit, In Vitro Biology, Lundbeck Research USA, 215 College Road, Paramus, NJ, USA

**Keywords:** ELISA, higher-throughput, LC–MS/MS, lipids, neuropathic pain

## Abstract

**Aim:**

Lipids such as prostaglandins, leukotrienes and thromboxanes are released as a result of an inflammatory episode in pain (central and peripheral).

**Methodology & results:**

To measure these lipids as potential mechanistic biomarkers in neuropathic pain models, we developed a higher-throughput LC–MS/MS-based method with simultaneous detection of PGE2, PGD2, PGF2α, LTB4, TXB2 and 2-arachidonoyl glycerol in brain and spinal cord tissues. We also demonstrate that the LC–MS/MS method was more sensitive and specific in differentiating PGE2 levels in CNS tissues compared with ELISA.

**Conclusion:**

The ability to modify the LC–MS/MS method to accommodate numerous other lipids in one analysis, demonstrates that the presented method offers a cost–effective and more sensitive alternative to ELISA method useful in drug discovery settings.

## Lay abstract

In humans, lipids carry out various functions such as energy production and storage, insulation, digestion and absorption and hormone production. Out of the several lipids, prostaglandins, thromboxanes and leukotrienes play a critical role in cardiovascular diseases, allergic reactions and inflammation. Thus, it is important to monitor their levels as potential mechanistic biomarkers to effectively diagnose and treat the underlying diseases. We have successfully used a highly specific and higher-throughput mass spectrometric method to quantify these lipids in brain cells as well as in brain and spinal cord tissues from rats (pain model) and compared the data obtained in the traditional ELISA.

**Figure 1.**
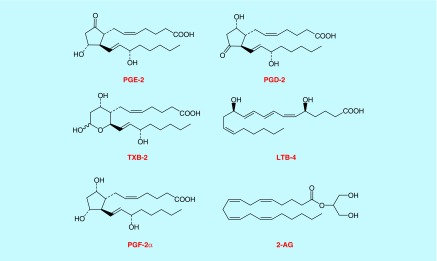
**Representative structures of lipids.** PGE2 and PGD2 are structural isomers of each other. 2-AG is an endocannabinoid known to be regulated in neuropathic pain. TXB2 and LTB4 are known proinflammatory mediators which play a significant role in nerve root dysfunction.

**Figure 2.**
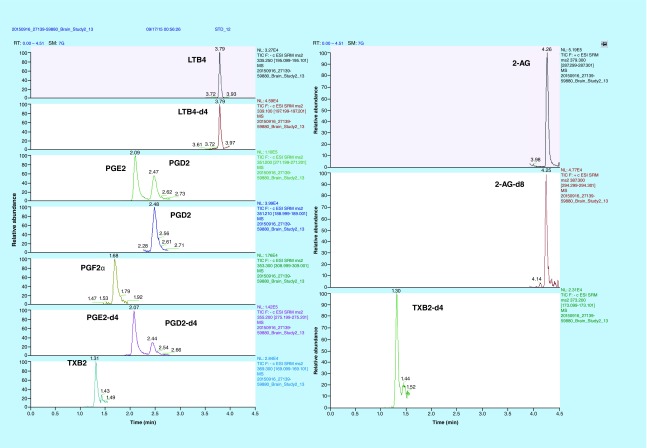
**Representative extracted ion chromatograms of lipids.** All lipids were base line resolved under <5 min with a gradient elution using 0.1% formic acid in MilliQ water and 0.1% formic acid in acetonitrile with a 0.5 ml/min flow rate and 20 μl injection volume. Except 2-AG, all lipids ionized in the negative ion mode. MRM transitions and retention times are listed in Table 1. MRM: Multiple reaction monitoring.

**Figure 3.**
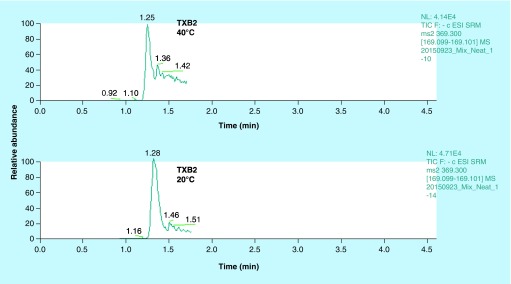
**Representative extracted ion chromatograms of TXB2.** We observed a distorted peak for TXB2 at 40°C column temperature as opposed to lower temperature of 20°C. TXB2 exists as anomers at higher temperatures; therefore it was imperative to reduce the column temperature for achieving a sharper peak.

**Figure 4.**
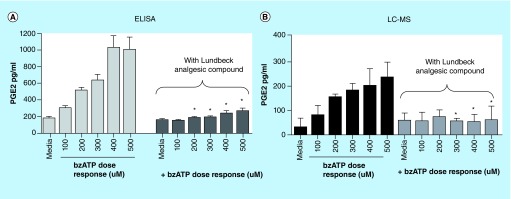
**PGE2 levels determined in *in vitro* microglial cell cultures.** As outlined in the methods section above microglia were cultured from 3-day-old Sprague-Dawley rat pups and treated with Lundbeck analgesic compound for 30 min with or without Bz-ATP for an additional 30 min. PGE2 levels were determined using a commercially available ELISA kit **(A)** or LC–MS/MS **(B)**. Data represented are mean ± SD from three separate experiments. High cross-reactivity of ELISA kit mentioned by the manufacturer shows approximately threefold higher levels of PGE2 compared with more specific LC–MS/MS analysis. *p < 0.05 when compared with bz-ATP-treated cells. BzATP: 2,3-O-(4-benzoylbenzoyl)-adenosine triphosphate; LC-MS/MS: Liquid chromatography tandem mass spectrometry.

**Figure 5.**
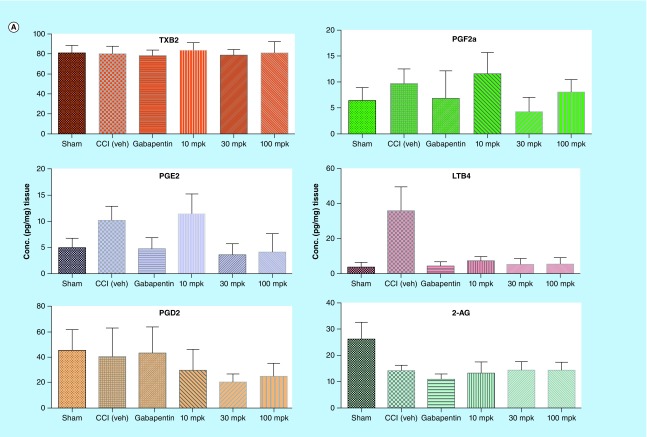

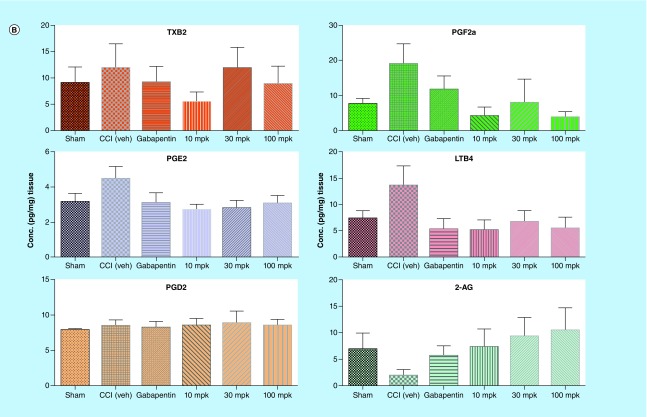
**Quantitation of lipids in CNS tissues from *in vivo* study using LC–MS/MS.** All the lipids were successfully quantified with acceptable recoveries and sensitivity using triple quadrupole mass spectrometer. Control rats (saline treated) and rats undergoing a chronic constriction injury of the sciatic nerve were treated with Lundbeck analgesic compound at 10, 30 and 100 mg/kg orally as, described in the methods section above. LODs and quantitation for the lipids are listed in Table 2. In brain **(A)**, significant changes were observed with PGE2 and LTB4 levels in CCI rats after treatment with analgesic compound when compared with CCI+Veh group. In spinal cord **(B)**, significant changes were observed with PGE2, PGF2α, LTB4 and 2-AG in CCI rats with Lundbeck analgesic compound compared against CCI+Veh treated animals. *p < 0.05 when compared against CCI+Veh. Data shown are mean ± SD from seven to ten animals per treatment group. CCI: Chronic Constriction Injury; veh: Vehicle.

## Background

Inflammation is the primary mechanism to an immune response upon microbial infection. Some inflammatory pathways are believed to play a pivotal role in pathogenesis of several neurodegenerative diseases such as Alzheimer's disease or Parkinson's disease as well as peripheral diseases such as rheumatoid arthritis and inflammatory bowel disease [1]. In inflammation and neuroinflammation, a common feature includes activated immune cells such as macrophages (liver) and microglia (brain). These induce COX enzymes resulting in oxidative stress as well as the production and release of cytokines and chemokines [2–4]. Studies have also shown that inflammatory pain can be characterized by release of lipid mediators from the COX pathway such as endocannabinoids, prostaglandins (PGs), thromboxanes, leukotrienes, among others from peripheral as well as CNS tissues such as brain and spinal cord [5, 6]. PGs such as PGE2, PGD2, PGF2α and Thromboxane B2 (TXB2) exhibit a wide variety of physiological and pathophysiological functions manifested by pain, fever and allergic reactions. Data from both preclinical and clinical studies substantiate the finding that PGE2 plays a critical role in exacerbating pain upon treatment with either peripheral or central stimuli [7–9]. Although most of these inflammatory molecules have a very short half-life in plasma (less than a minute), they are much more stable in brain and spinal cord tissues which allows quantitation of the intact molecules in both *in vitro* and *in vivo* matrices [10,11]. Thus the first goal of this study was to develop a LC–MS/MS method to simultaneously quantify these lipids as potential mechanistic biomarkers in an inflammatory disease animal model and using a neuropathic pain model for evaluating feasibility. The second goal was to compare the LC–MS/MS method against the more commonly used ELISA.

For more than two decades, ELISAs have been routinely used for quantifying protein or lipids in both *in vitro* and *in vivo* matrices. Although the commercially available ELISA kits have been fully validated for their LODs, linearity, specificity or cross reactivity, they do suffer from several other drawbacks like antibody specificity toward the protein of interest, cost and stability issues of the kits. Although ELISA can measure low concentrations of lipids with minimal sample preparation, cross-reactivity between isomeric species (PGE2, PGE1, PGD2, among others, which have same molecular weight but different structural configuration) cannot be completely eliminated leading to a higher false-positive rate compared with LC–MS/MS analyses [12]. Thus one of the goals of this study was to compare results for PGE2 between ELISA and LC–MS/MS.

Several analytical methods have been developed and published in literature for absolute quantification of lipids in biological matrices. These include traditional-flow LC–MS/MS [13] as well as nano-LC–MS/MS [14]. Although nano-LC–MS/MS offers several advantages such as very low sample volume requirements, low consumption of mobile-phase solvents and higher sensitivity, it is not commonly utilized due to the need for more complex instrumentation requiring additional investment and tedious sample handling procedures. Triple quadrupole LC–MS/MS is the preferred method for quantifying prostanoids, endocannabinoids and their metabolites. A recent study utilized online SPE as a rapid sample cleanup procedure for quantifying PGE2, PGD2 and TXB2 in different *in vitro* assay systems to determine the effects of COX-2 inhibitors [15]. Schmidt *et al*. validated an LC–MS/MS method for quantifying PGE2 and PGD2 in rat microdialysis samples with LLOQ of 25 pg/ml and 50 pg/ml, respectively [16]. Likewise, PGE2 and PGD2 were measured using a validated method from Cao *et al*., in human lung epithelial cell line A549 and the mouse macrophage cell line RAW 264.7 [17]. Although recent studies quantified LTB4 in human plasma or sputum specimens [18,19], their limits of quantitation were 0.2 ng/ml compared with 0.078 ng/ml observed in our study. Although relatively simple liquid–liquid extraction procedure was used in both studies, a more complex SPE has also been used for extracting lipids from brain tissues which require extensive sample cleanup [20,21]. Thus, considering the need for an assay with a significant capacity in drug discovery phase, our goal was to develop a reliable higher-throughput LC–MS/MS method for quantifying these lipids in a single injection with acceptable and reproducible recoveries.

As there are less specific requirements by the US FDA for validating bioanalytical methods for biomarker quantification, the presented data herein offer a great advantage of being a fit-for-purpose higher-throughput LC–MS/MS method for quantifying lipids in a drug discovery or early drug development setting. To our knowledge this is the first study to monitor multiple lipids in both positive and negative polarity in a single injection in less than 5-min run time without compromising the sensitivity. We also show the utility of our method for measuring lipids in both *in vitro* microglia culture and *in vivo* rat brain and spinal cord tissues and compare the results with the standard ELISA method used for PGE2 from *in vitro* samples. We would also like to mention that the presented method can also be applied to other sample types such as plasma or cerebrospinal fluid for quantifying lipids as biomarkers.

## Methods

### Chemicals & reagents

Authentic reference standards for PGE2, PGD2, PGF2α, LTB4, TXB2 and 2-AG and their deuterated analogs (PGE2-d4, PGD2-d4, LTB4-d4 and TXB2-d4) were obtained from Cayman Chemical (MI, USA) except for 2-AG whose deuterated analog (2-AG-d8) was obtained from Abcam (MA, USA). Hepes balanced salt solution and Dulbecco's modified Eagle's medium (DMEM) were purchased from Gibco, Life Technologies (NY, USA). Fetal bovine serum (FBS) and all other cell culture reagents were obtained from Atlanta Biologicals (GA, USA). LC–MS/MS grade acetonitrile and methanol were obtained from Fisher Scientific (NJ, USA). Formic acid was obtained from Sigma-Aldrich (MO, USA). Deionized water was produced in-house using a Milli-Q Gradient water purification system (MA, USA).

### Method development: LC–MS/MS optimization

The LC–MS/MS system consisted of triple quadrupole mass spectrometer operated in multiple reaction monitoring mode (TSQ Quantum, Thermo Scientific, CA, USA). A heated ESI was operated in negative mode for all analytes except 2-AG, where positive ESI mode was used. The mass spectrometer was optimized for various parameters applicable to all the analytes. The spray voltage was set at 2000 kV in negative mode and 4500 kV in positive mode, vaporizer temperature was 450°C, cycle time of 0.3 s, capillary temperature was set at 325°C, sheath gas and AUX gas pressures were set at 40 and 20 psi, respectively. The analytes were run in full scan mode to determine their molecular ion peaks and collision activated ionization was performed on the molecular ion peak in MS/MS mode. The tube lens and collision energies were optimized for each analyte individually. A divert valve was used to acquire data between 1 and 4.6 min to reduce unwanted saturation of the detector. The molecular ion and fragment ion peaks, tube lens and their collision energies are shown in Table 1. Data were processed using Xcalibur software 3.0.63.

Chromatographic separation was performed on an Acquity UPLC system from Waters (MA, USA). Analytes were resolved on a Kinetex C18 (2.1 × 50 mm) column with an internal particle size of 2.6 μm (Phenomenex, CA, USA). In order to achieve shorter run times, a linear gradient with 0.1% formic acid in Milli Q Gradient water and 0.1% formic acid in acetonitrile was employed. To achieve efficient resolution, the LC flow was set at 0.45 ml/min with the following gradient: 0–3 min 30% B, 3–3.65 min 30–90% B, 3.65–4.8 min 90% B, 4.8–5 min 90–30% B. Total run time was 5 min. Injection volume of samples was 20 μl. Column temperature was set at 20°C.

### Standard preparation

Standards were obtained as 100 μg/ml or 1 mg/ml solutions. From these 1 μg/ml working stock solutions were prepared in acetonitrile and stored at -80°C. For *in vitro* samples, calibration curves were generated in DMEM containing 2% FBS from 0 to 10 ng/ml spiked with 10 ng/ml deuterated analog which served as internal standard (IS). For *in vivo* samples, the blank brain and spinal cord tissue homogenates were diluted ten times to reduce endogenous lipid level and calibration curves were generated from 0 to 10 ng/ml containing 10 ng/ml of deuterated IS.

### Recovery, matrix effect & LOQ

Recoveries of the analytes were monitored by comparing peak area responses obtained from reference standard and deuterated analog (IS) spiked sample before extraction and postextraction at concentrations of 0.156, 1.25 and 5.0 ng/ml in DMEM medium for *in vitro* samples. Due to the presence of endogenous lipids in brain and spinal cord tissues, *in vivo* recoveries were determined by spiking 0.2 and 1.0 ng/ml of deuterated IS for each analyte in brain and spinal cord tissue homogenates, respectively. Matrix effect was determined by comparing the peak area responses between postextracted and neat sample (prepared in mobile phase). Linearity was determined by monitoring the peak area response from 0 to 10 ng/ml for both *in vitro* and *in vivo* samples. LOD was determined as an S/N ratio of 3:1 and limit of quantitation was based on an S/N of 10:1 for all the analytes.

### Carry-over assessment

Carry-over from injection needle was assessed by injecting blank samples after the high calibrator standard (10 ng/ml). Peak area of blank sample was compared with the peak area of the LOQ samples for each analyte. The blank sample peak area should be less than 20% of that of LOQ samples to avoid any sample carry-over.

### Isolation & culture of primary rat microglia

Primary mixed glial cultures were prepared from 3-day-old Sprague–Dawley rats (Charles River, NY, USA) as described previously [22]. Cerebral cortices were removed of meninges and minced in Hepes balanced salt solution. Cells were dissociated in a complete culture media. The complete culture media consisted of DMEM-GlutaMax containing 4.5g/l D-Glucose, 10% FBS (Atlanta Biologicals, GA, USA) and penicillin/streptomycin. Cells were plated into flasks containing 25 ml culture medium at a density of one brain per T150 flask (BD Falcon, NY, USA). Cells were maintained at 37°C in a 5% CO_2_ humidified atmosphere. After 2 days the flasks were tapped gently to remove cell debris, media removed and replaced with fresh media. Cultures were grown for 10–14 days at which time the microglia were harvested by tapping the flasks and collecting the microglia-enriched containing medium. Microglia were pelleted by centrifugation (1100 rpm, 5 min), resuspended in culture media and plated at desired density in poly-d-lysine-coated plates (1 × 10^5^ cells/200 ml in a 48-well plate) (Becton Dickinson BioCoat, MA, USA). Purity was assessed by labeling with the microglial maker CD11b, which identified 95% of cells as microglia.

### Treatment of microglia with analgesic compound & Bz-ATP followed by ELISA for PGE2 quantitation

Individual wells containing microglial cells were treated with 1 μM Lundbeck propriety analgesic compound, followed by Bz-ATP (known to induce inflammatory response) from 0 to 500 μM for up to 30 min and samples were then collected and stored for PGE2 analysis by ELISA. Microglia were plated in 48-well poly-d-lysine-coated plate at a density of 1 × 10^5^ cells/200 ml in DMEM media containing 10% FBS and were cultured overnight. Next day, media were replaced with DMEM containing 2% FBS. Cells were treated with different concentrations of Lundbeck analgesic compound (0–500 μM) for 30 min and followed by Bz-ATP treatment at final concentration of 300 μM. DMSO was utilized as a diluent. Supernatants were harvested 30 min after Bz-ATP treatment and assayed for PGE2 secretion. PGE2 levels in supernatants were determined by PGE2 EIA kit (Cayman Chemical Co, MI, USA) according to the manufacturer's protocol.

### LC–MS/MS sample preparation

#### In vitro samples

Microglial cell culture supernatants (90 μl) were added to 10 μl of an IS mixture containing 100 ng/ml of deuterated PGE2, PGD2, TXB2, LTB4 and 2-AG prepared in acetonitrile. Samples were protein precipitated with 300 μl each of acetonitrile and ethyl acetate. After a brief vortexing (∼30 s), samples were centrifuged at 3300 rpm for 20 min at 4°C. The supernatant were collected in a clean plate, dried under N_2_ gas at 40°C and reconstituted in 125 μl 30% acetonitrile solution containing 0.01% formic acid in water. Twenty microliter was injected onto the LC column.

### Animal treatment & *in vivo* sample (brain & spinal cord) preparation

We tested the utility of our LC–MS/MS method in a rat pain study in chronic constriction injury model of sciatic nerve using a Lundbeck propriety compound (10, 30 and 100 mg/kg oral administration) which was developed as an analgesic. All studies were conducted in strict accordance with the recommendations set forth in the Guide for the Care and Use of Laboratory Animals (published by NAP, 2011) and Institutional Animal Care and Use Committee at Lundbeck Research, USA. Male Sprague Dawley rats (Harlan Laboratories, TX, USA) were used in the study. Animals were divided into six different groups of ten animals per treatment group: saline, Vehicle, gabapentin (100 mg/kg) and analgesic compound (10, 30 and 100 mg/kg), respectively. Upon receipt, rats were housed three per cage in standard cages. Rats were allowed to acclimate up to 1 week prior to surgery. All rats were examined and weighed prior to initiation of the study to assure adequate health and suitability. During the course of the study, 12-h light/dark cycles were maintained. The room temperature was maintained between 20°C and 23°C with a relative humidity maintained around 50%. Chow and water were provided *ad libitum* for the duration of the study. All testing was performed during the animals’ light cycle phase. Rats were single housed after surgery. This surgery was performed according to [23]. Only the lumbar spinal cord segments were used in this study.

After completion of accessing the presence of analgesic activity, brain and spinal cord tissues were harvested (within 15 min of testing) following euthanasia by CO_2_ inhalation. Samples were stored at -80°C. Brain was homogenized in 3× and spinal cord in 4× w/v in an aqueous MSD lysis buffer (MesoScale Discovery, MD, USA). The homogenates were used for lipid extraction as described above (*in vitro* sample preparation).

### Data analysis

For determining the concentration of unknown samples, ratios of analyte peak area and IS peak area (y-axis) were plotted against concentration (x-axis) and calibration curves for each prostaglandin were generated by least square regression analysis with 1/X^2^ as a weighting factor. Statistical significance was determined by Student's *t*-test with p < 0.05 using GraphPad Prism (6.0). The values are presented as mean ± SD and n = 4–5 samples per group for *in vitro* assays and n = 8–10 animals for *in vivo* study.

## Results & discussion

### Chromatograms of various lipids

The chemical structures of various lipids measured in this study are shown in Figure 1. The mass spectrometer optimization parameters are listed in Table 1. All six analytes, namely PGE2, PGD2, PGF2α, LTB4, TXB2 and 2-AG and their deuterated ISs were resolved under a LC run time of <5 min (Figure 2). To our knowledge this is the first study to achieve chromatographic separation of five lipid analytes ionizing in negative ESI mode and one in positive ESI mode in a single injection. As PGE2 and PGD2 are isobars (different structure with same molecular mass) as well as geometrical isomers (similar fragmentation pattern), we had to baseline resolve them in order to obtain sufficient confidence in quantitation. A previously published study required LC run time of more than 30 min to resolve PGE2 and PGD2 by HPLC [24]. However, our method efficiently resolved PGE2 and PGD2 under isocratic conditions (30% B) in <3 min eluting at 2.08 and 2.47 min, respectively (Figure 2). We think this observation could be due to a different UPLC column which we used (Kinetex C18) as opposed to Luna C18 column used by Brose *et al*. [24]. We also did a temperature-peak shape curve to evaluate if column temperature could affect the baseline resolution of these two isomers (discussed in the next paragraph). Also to reduce cycle time for the instrument, the data were acquired in scheduled MRM mode after determining the retention time of the analytes. As shown in Figure 2, the retention times for PGE2/PGE2-d4 were 2.08 min, PGD2/PGD2-d4 were 2.47 min, PGF2α was 1.68 min, TXB2/TXB2-d4 were 1.30 min, LTB4/LTB4-d4 were 3.79 min and 2-AG/2-AG-d8 were 4.25 min, respectively.

### Effect of temperature on TXB-2 chromatography

We had initially set the column temperature at 40^°^C to achieve sharp peaks. However, TXB2 is known to exist in two distinct anomeric forms (differs in configuration at the anomeric carbon atom) [25]. So it is important to separate these two anomers in the LC run. They can be separated by varying the column temperature where lower temperatures favor their resolution [25]. Thus we were able to achieve clean sharp peak for TXB2 (Figure 3) without compromising the peak shapes for all other analytes. For this reason, we used a 2.6 μ column instead of 1.8 μ to prevent increased column back pressures.

### Recovery, matrix effect & LOQ

A variety of different solvent combinations were tested for obtaining sufficient and reproducible analyte recoveries. As a general guide for developing bioanalytical methods for biomarker measurements [1], the analyte recoveries are not always expected to be 100%, but rather they should be consistent, precise and reproducible. For this purpose, specifically, various solvent mixtures of chlorofom:methanol:water, hexane:ethyl acetate, acetonitrile:methyltert-butyl ether, acetonitrile:ethyl acetate or hexane:Isoproply alcohols were tested for individual lipids at 0.156, 1.0 and 5.0 ng/ml for *in vitro* or 0.2 and 1.0 ng/ml concentrations for *in vivo* samples. The analyte recoveries ranged from 50 to 80% for all analytes in cell culture medium, brain and spinal cord tissues using only 1:1 acetonitrile:ethyl acetate mixture. The recoveries were much lower with other solvent mixtures (data not shown). Thus, we decided to use 1:1 acetonitrile:ethyl acetate as the solvent combination for all the data presented in this study. IS recoveries were also in the same range (50–80%) as deuterated analogs of analyte standards were spiked in the matrix.

In general, if matrix effects were to impact analysis, it would be better to have minimum positive (ion enhancement) than negative (ion suppression) for matrix interferences. Although there are various ways to control matrix effects (e.g., using a synthetic surrogate matrix or charcoal-stripping approach [26], it is not always possible to minimize matrix effects for multiplex assays in a drug discovery setting. In our study the matrix effects were approximately ±10% at the 1.25 and 5.0 ng/ml concentrations. The matrix effects for all analytes were 22–56%, except TXB2 (>200%) at the 0.156 ng/ml concentration in *in vitro* specimens. The matrix effects were found to be 10–40% for *in vivo* brain and spinal cord samples when spiked with deuterated analogs (data not shown). Incorporation of these deuterated analogs as IS were able to correct for matrix effects by normalizing the peak areas at retention times corresponding to their respective lipid standards.

The limit of detection was based on 3:1 S/N ratio for the analytes in spiked matrix sample. The limit of quantitation was based on a 10:1 S:N ratio. Based on these criteria, the LOD and LOQ are presented in Table 2. The numbers are comparable to the ones reported in the literature [17].

There was negligible sample carry-over for all analytes as the blank peak areas were <1% of the LOQ samples.

### Comparison of LC–MS/MS data versus ELISA data for PGE2

An important aspect of this study was to build a higher-throughput LC–MS/MS method as an alternate to the costly and to a certain degree as mentioned earlier nonspecific ELISA platform used in lipid quantification. Although ELISA is routinely used for a large number of analyses, they come at an analytical cost with detection of antibodies carrying some cross reactivity toward other lipids [27]. As compared with PGE2 levels detected from ELISA upon Bz-ATP treatment shown in Figure 4A, the LC–MS/MS assay produced approximately two to threefold lower PGE2 concentrations (Figure 4B) in the same *in vitro* treatment suggesting likely increased specificity in PGE2 detection with analyte recoveries being very similar. We also compared PGE2 standard from the ELISA kit against chemical PGE2 on LC–MS/MS to verify PGE2 standard provided in the ELISA kit. Although they should have had similar responses in LC–MS/MS assay, the response for ELISA kit provided PGE2 standard was four- to fivefold lower than the chemical PGE2 standard we used. The observed difference in the response could be due to a formulation differences of PGE2 provided in ELISA kit as opposed to any differences attributed to LC–MS/MS method. Nevertheless, the observed fold-differences in PGE2 levels between various doses of Bz-ATP treatment alone and Bz-ATP+analgesic compound treatments on primary rat microglia were comparable between ELISA and LC–MS/MS platforms (Figure 4A vs Figure 4B). Although these results indicate that still ELISA can be used as a screening tool, the LC–MS/MS method provides improved specificity, enabling the method to be utilized not only as a screening tool, but a confirmatory tool in lipid profiling for potential biomarker support. In this study, the ELISA and LC–MS/MS comparisons were only made with *in vitro* samples with respect to PGE2. However, we also wanted to investigate the utility of the LC–MS/MS method for profiling *in vivo* lipids utilizing CNS tissues (brain and spinal cord). As evident from the analytical data described in greater detail in next paragraph, this method has the ability to detect multiple lipids at low picomolar levels in CNS tissues obtained from animals with inflammation-mediated pain. With appropriate method optimization, the authors feel this method could be further extended with minimal effort for profiling additional lipid analytes as well as quantifying lipids in peripheral tissues of inflammatory models. It would be challenging to create such a versatile multiplexing method using an ELISA platform, given some of the limitations discussed earlier.

### Application of LC–MS/MS platform for *in vivo* studies

As one of the goals of this study was to quantify lipids as potential mechanistic biomarkers in inflammation mediated pain models, we measured lipid levels in CCI (Chronic Constriction Injury) rat brain and spinal cord tissues. We were able to measure all key lipids as depicted in Figure 1 in sham as well as CCI cohorts with underlying inflammation mediated pain treated with either vehicle or analgesic compound (Figure 5A & B). The observed range of lipid analyte levels that were quantified across both brain and spinal cord tissues suggests this LC–MS/MS assay platform is quite capable of quantifying multiple lipids in *in vivo* matrices with minimal matrix interference. In addition, this multiplex method with higher-throughput capability and flexibility for modification is very amenable to drug discovery settings to assay lipids in both *in vitro* and *in vivo* matrices. We have clearly demonstrated here by quantifying six different analytes (PGE2, PGD2, PGF2α, TXB2, LTB4 and 2AG) simultaneously in both rat brain and spinal cord tissues with only 5-min analytical run time (Figure 5A & 5B).

## Conclusion

In this publication, we have described an LC–MS/MS method capable of simultaneously quantifying inflammation mediated lipid analytes, PGE2, PGD2, PGF2α, TXB2, LTB4 and 2AG in both *in vitro* and *in vivo* matrices. To the best of our knowledge this is the first demonstration of a multiplexing analytical method in a higher-throughput mode for quantifying multiple lipids analytes with a 5-min run time. The analyte separation was accomplished using a generic mobile phase system for each injection. Though the LC–MS/MS method is comparable to traditional ELISA platforms quantifying lipids, the observed fold-changes of lipid analyte detection such as PGE2 in rat microglial cells described here offers the potential for detecting as well as quantifying additional lipid analytes (Figure 4). In addition, the LC–MS/MS method detected much lower levels of PGE2 compared with ELISA in same *in vitro* samples (Figure 4), suggesting increased specificity with respect to detection of individual lipid analytes. This key difference could be potentially attributed to one of the limitations of ELISA related to reduced specificity in antibody mediated detection with certain nonspecific binding contributing to lipids detection in general as the observed increased PGE2 levels compared with LC–MS/MS analyses in our hand. Nevertheless, the ability to accommodate faster analysis time of multiple lipid analytes (multiplex mode) with increased specificity, flexibility and lower cost makes the higher-throughput LC–MS/MS-based assay platform described here more conducive for potential lipid biomarker efforts in drug discovery settings.

## Future perspective

Evaluation of endogenous molecules as mechanistic biomarkers is a potential strategy pursuing disease pathologies. Here we have pursued endogenous lipid molecules such as prostaglandins, thromboxane B2, leukotriene B4 and 2-arachidonoyl glycerol (endocannabinoid) as potential CNS biomarkers of inflammatory mediated neuropathic pain. We utilized LC–MS/MS-based methodologies and have developed a cost–effective higher-throughput method capable of simultaneous detection of lipids with increased specificity and quantification in different tissue matrices. This method is more amenable to drug discovery settings than traditional ELISA platforms with specificity limitations used in detection antibodies as well as cost of multiplexing ELISA platforms. Therefore, we believe the future utility of this method in evaluating these lipid molecules as well as other lipids as mechanistic probes and/or biomarkers across various inflammatory disease platforms preclinically. In addition to drug discovery application of this method, we believe that this LC–MS/MS method has the potential to be used for large sample sizes in evaluating clinical diagnostic/translational following a more rigorous validation in numerous biological matrices such as serum, whole blood, tissue homogenates and cerebrospinal fluid.

**Table 1.** **Multiple reaction monitoring parameters of lipids: all the lipids except 2-AG were ionized in negative electrospray ionization mode on Thermo Quantum Triple Stage Quadrupole mass spectrometer.**

**Analyte**	**Precursor ion *m/z***	**Fragment ion *m/z***	**MRM collision energy**	**Retention time (min)**	**Time window (min)**	**Tube lens**	**Polarity**	**Trigger**
TXB2	369.3	169.1	19	1.29	0.80	62	Negative	100
TXB2-d4	373.2	173.1	20	1.29	0.80	47	Negative	100
PGF2	353.3	309.0	21	1.69	0.60	90	Negative	100
PGE2/PGD2	351.2	271.2	19	2.30	1.00	45	Negative	100
PGE2-d4	355.2	275.2	17	2.30	1.00	43	Negative	100
LTB4	335.3	195.1	18	3.77	0.60	60	Negative	100
LTB4-d4	339.1	197.2	18	3.77	0.60	77	Negative	100
2-AG	379.3	287.3	14	4.25	0.80	59	Positive	100
2-AG-d8	387.3	294.3	14	4.25	0.80	62	Positive	100

Precursor and fragment ions, collision energies and tube lens were optimized during method development. A retention time window was set to acquire data around each lipid's expected retention time to minimize matrix effect and increase sensitivity.

MRM: Multiple reaction monitoring.

**Table 2.** **LOD and LOQ of lipids: determination of LOD was based on an S/N ratio of 3:1 and LOQ was based on an S/N ratio of 10:1.**

**Analyte**	**Retention time (min)**	**LOD (ng/ml)**	**LOQ (ng/ml)**
TXB2	1.29	0.019	0.039
PGF2	1.69	0.019	0.078
PGE2	2.08	0.009	0.039
PGD2	2.47	0.009	0.039
LTB4	3.78	0.019	0.078
2-AG	4.25	0.078	0.156

The LODs and LOQs observed in the current study are comparable to the published data for all of the lipids.

**Executive summary**

**Background**

Development of a higher-throughput LC–MS/MS-based assay for quantitation of endogenous lipids as potential mechanistic biomarkers using inflammatory mediated neuropathic pain model, and its comparison against ELISA.

**Results**

We successfully resolved six endogenous lipids under 5-min separation run time with acceptable recoveries and sensitivity using a liquid–liquid extraction method. These lipids were quantifiable in multiple matrices. The absolute PGE2 concentrations determined *in vitro* were two- to threefold lower in LC–MS/MS compared with enzyme-linked immunosorbent assay determination. No significant differences were observed in PGE2 (fold-change) between Bz-ATP-stimulated or nonstimulated microglial cells when treated with the analgesic compound.

**Conclusion**

To best of our knowledge, this is the first demonstration of a higher-throughput LC–MS/MS method for quantifying multiple lipids in a number of CNS matrices as potential biomarkers to be used in a drug discovery mode.
